# Women's fertility desires and contraceptive behavior in three peri-urban communities in sub Saharan Africa

**DOI:** 10.1186/s12978-016-0118-z

**Published:** 2016-02-12

**Authors:** Funmilola OlaOlorun, Assefa Seme, Easmon Otupiri, Peter Ogunjuyigbe, Amy Tsui

**Affiliations:** Department of Community Medicine, College of Medicine, University of Ibadan, Queen Elizabeth Road, P.M.B. 5116, U.C.H. Ibadan, Nigeria; Department of Reproductive Health & Health Service Management, School of Public Health, Addis Ababa University, Addis Ababa, Ethiopia; Department of Community Health, Kwame Nkrumah University of Science and Technology, Kumasi, Ghana; Department of Demography and Social Statistics, Obafemi Awolowo University, Ile-Ife, Nigeria; Department of Population, Family & Reproductive Health, Johns Hopkins Bloomberg School of Public Health, Baltimore, MD USA

**Keywords:** Fertility desires, Contraceptive behavior, Peri-urban, Sub-saharan Africa

## Abstract

**Background:**

Fertility desires and contraceptive behavior often change over time. This study examined the influence of change in fertility desires on change in modern contraceptive use over time in three peri-urban communities in sub-Saharan Africa.

**Methods:**

This multi-site study includes baseline and follow up data from 3 sites in the Family Health and Wealth Study. Following a census in each site, a probability sample of at least 500 households was obtained. Generalized linear models were employed.

**Results:**

Modern contraceptive use increased in Ipetumodu, Nigeria (29.4 % to 36.7 %), but declined slightly in Sebeta, Ethiopia (66.9 % to 61.3 %) and Asawase, Ghana (12.6 % to 10.8 %). Across sites, at baseline and follow up, women who wanted no more children reported more contraceptive use, compared with those who wanted more children, and were more likely to shift to being contraceptive users in Ipetumodu [aOR(95 % CI):1.55 (1.07,2.26)].

**Conclusions:**

Women’s fertility desires influenced their contraceptive behavior, although there were cross-site differences. Changing contraceptive demand and program factors will be important to enable peri-urban women to frame and act on their fertility desires.

## Background

Women’s fertility motivations, desires and intentions, as well as how these constructs are related to fertility behavior, such as contraceptive use, have been studied by researchers from different geographical settings and varying socio-cultural contexts. Fertility desires are personal preferences and do not translate into behavior until after they have been transformed into intentions, described as, “conscious commitments to act in a certain way or to try to achieve a certain goal at some future time” [1]. Different types of desires and corresponding intentions have been described as predictors of fertility: timing of child bearing and number of children [1].

Researchers have shown that the proportion of women who want no more children can strongly predict contraceptive prevalence and fertility rates [2, 3], suggesting that women who want no more children may in fact be very different, and more motivated to attain their fertility goals, when compared with women with other fertility desires. However, cross-sectional studies conducted in Burkina Faso, Ghana and Kenya [4], two-time point interviews of women in Honduras [5], and a longitudinal study conducted in Morocco [6] suggest that women’s reports of their fertility motivations, desires and intentions are often inconsistent with their contraceptive behavior.

Research from both developed [7–9] and developing [4–6] world settings suggests that women may be ambivalent about whether or not to get pregnant, and this affects their contraceptive decision making, as well as choice of method. In a study conducted in Malawi, contraceptive use was shown to be lower among women in polygynous unions [10]. However, fertility preferences of husbands or wives did not differ substantially in monogamous or polygynous unions, possibly because these preferences were largely influenced by attitudes, beliefs, and societal norms, developed over time [10].

The most recent Demographic and Health Surveys from Ethiopia, Ghana and Nigeria [11–13] reveal that fertility remains high and contraceptive use low across these three contexts. Total fertility rates were 4.8, 4.0, and 5.5 births among all women of reproductive age in Ethiopia, Ghana and Nigeria respectively. Modern contraceptive use by currently married women ages 15–49 years was much higher in Ethiopia (27.3 %) than Ghana (16.6 %) and Nigeria (9.8 %). The diversity of experiences among women in very similar, yet distinctly different Sub-Saharan Africa (SSA) contexts may be a reflection of the strong programmatic attention to and use of community health extension workers in distributing family planning commodities in Ethiopia [14], but weak and indifferent health care delivery systems for contraceptive service delivery in Ghana and Nigeria.

Our study aims to add to the growing body of literature on fertility desires and contraceptive use from high fertility settings. Many studies are cross-sectional and focus on women who live in urban and/or rural settings. We use panel data to focus on women in peri-urban settings who share characteristics with both urban and rural contexts. More specifically, this paper answers the following research questions:

(1) Do women with different fertility desires differ in their contraceptive use? We hypothesize that if indeed fertility desires are strong predictors of contraceptive use, then, women who want no more children should report more contraceptive use at both time points compared with women who want more.

(2) Is the change in modern contraceptive use over time the same for women who want more and those who want no more children? We hypothesize that women who want more children at baseline will report less contraceptive use at follow up. Similarly, women who want no more at baseline will report more contraceptive use by follow up. Furthermore, we hypothesize that the change in contraceptive use observed will be similar in both groups, although in opposite directions.

(3) Does the relationship between fertility desires and modern contraceptive use over time remain, even after adjusting for socio-demographic variables? We hypothesize that the relationship will persist after adjusting for potential confounders.

## Methods

The data come from the Family Health and Wealth Study, a multi-site longitudinal community-based study which recruited at least 500 households from each of nine peri-urban communities in 8 countries (Ethiopia, Ghana, Malawi, Nigeria, Uganda, Egypt, India and China). Households have been interviewed at two time points (2009/2010 and 2011/2012). In each site, a census of households in the selected peri-urban areas was followed by a systematic selection of a probability sample of households with a resident married (or living as married) couple. If a selected household did not have an eligible woman aged 15–44 years and an eligible male partner aged 20–59 years, or the eligible couple did not consent to participate in the study, another eligible couple was selected from the same or an adjacent household. Women who were not married or who did not live with their partners were not eligible to be enrolled.

At baseline, the survey included five modules – household, male, female, household wealth and physical assessment modules. The household head served as the respondent for the household and wealth modules. Face-to-face interviews were conducted with men and women separately to obtain information such as socio-demographic and reproductive health characteristics. At follow-up, similar modules were administered but some questions were modified to refer to baseline information.

Both at baseline and follow up, women were asked about their fertility desires and current contraceptive use. The outcome of interest was change in modern contraceptive use over the two rounds. The key independent variable was a woman’s fertility desire, derived from her response to the question, “Would you like to have (more) children (than you have now)?” The response options were “yes”, “no” and “don’t know”. Women who reported “don’t know” were few and grouped with women who said “no” after a separate analysis revealed no statistically significant difference in the two groups’ characteristics. Thus, the binary variable derived was coded “1” for women who wanted no more children and “0” for those who wanted more.

Potential confounders that were included in the models were age, parity, education, polygyny, and wealth. Age was categorized as <25 years, 25–34 years and 35–49 years. Parity was defined as the number of children ever born and grouped as 0–1, 2–3 and 4 or more. Educational level was grouped as no formal, primary, secondary and tertiary education. For Ghanaian women, the secondary and tertiary education categories were collapsed due to the small percentage of women with tertiary education. Polygyny was dichotomized as being in a polygynous union or not. Wealth scores were constructed through a principal component analysis of items measuring ownership of different household assets, housing construction quality and sanitation facilities. The scores were then grouped into wealth quintiles.

The respective samples for the current analyses come from women interviewed at baseline from three peri-urban communities: Sebeta, Ethiopia (*n* = 998); Asawase, Ghana (*n* = 800) and Ipetumodu, Nigeria (*n* = 776). At follow up, there were 741 women in Sebeta (74 % of baseline cohort); 623 women in Asawase (78 % of baseline cohort); and 492 women in Ipetumodu (63 % of baseline cohort). Reasons for loss to follow up were mostly movement outside of the study sites. Other reasons were refusal to participate, divorce and death. Women with information missing on the outcome variable were excluded from the present analysis (less than 3 % of sample per site). During exploratory data analysis, continuous variables were summarized using means and medians while categorical variables were tabulated. Variables associated with contraceptive use at the level of *p* < 0.1 and those conceptualized to confound the association between fertility desires and contraceptive use were retained in the final models. The other three peri-urban communities in SSA were excluded from the present analyses because half or more of the baseline sample were lost to follow-up. The study design and instruments for the study sites in Egypt, India and China differed from those of the SSA sites, preventing cross-site comparison.

Propensity score analysis was used to address the potential bias from loss to follow up. First, a logistic regression model was used to estimate the probability of being followed up, using observed baseline variables as predictors [15, 16]. After assessing fit and suitability of the included covariates, predicted probabilities of follow-up were generated for each respondent. The inverse of that probability of follow up was then used to weight the respondents for the final generalized linear models (GLM). The GLM, with a binary logistic outcome for contraceptive use at baseline or follow up, with assumptions about data structure and intra-class correlations specified as needed, produces unbiased estimates of change in modern contraceptive use. All analyses were conducted using Stata (version 12; StataCorp., College Station, TX, USA).

Ethical approval for the study was obtained from the site-specific institutional ethical review committees of Addis Ababa University, Ethiopia; Kwame Nkrumah University of Technology, Kumasi, Ghana and Obafemi Awolowo University, Ile-Ife, Nigeria. All participants provided verbal informed consent prior to each interview.

## Results

On average, women in the sample were 29.0 ± 6.3 years; 33.7 ± 6.3 years; and 33.3 ± 10.1 years in Sebeta, Asawase and Ipetumodu respectively at baseline. In Sebeta, Asawase, and Ipetumodu, 55.8 %, 38.2 % and 62.9 % women respectively had at least secondary level education. Women had an average of 2.3 ± 1.7 children in Sebeta; 3.3 ± 2.0 children in Asawase; and 2.9 ± 1.7 children in Ipetumodu.

Women seen at follow up differed from those lost to follow up on different characteristics across sites. In Sebeta and Asawase, women seen at follow up had significantly higher parity, compared with those lost to follow up. In Sebeta and Ipetumodu, women seen at follow up were less likely than those not seen to have considered divorcing from their current partner. Women retained in the Sebeta sample had lived there longer than those lost to follow up.

Table 1 shows the characteristics of women who used modern contraception at baseline and follow up by socio-demographic characteristics and site. The magnitude and direction of change in modern contraceptive use between baseline and follow up differed across the three sites, increasing in Ipetumodu (29.4 % to 36.7 %), but slightly reducing in Sebeta (66.9 % to 61.3 %) and Asawase (12.6 % to 10.8 %).

**Table 1:** Percent using modern contraception by fertility desire and selected background characteristics

	Sebeta, Ethiopia		Asawase, Ghana		Ipetumodu, Nigeria	
	*N* = 986 Baseline (%)	*N* = 741 Follow up (%)	*N* = 784 Baseline (%)	*N* = 623 Follow up (%)	*N* = 761 Baseline (%)	*N* = 479 Follow up (%)
Current use of modern contraception [*n* (%)]						
Yes	660 (66.9)	454 (61.3)	99 (12.6)	67 (10.8)	224 (29.4)	176 (36.7)
No	326 (33.1)	287 (38.7)	685 (87.4)	556 (89.3)	537 (70.6)	303 (63.3)
% of modern contraceptive users:						
Fertility desires						
Want more	63.9	64.1	12.0	12.5	25.8	26.3
No more	71.3^a^	71.9^a^	13.9	13.0	37.2^b^	39.0^b^
Education						
No formal	70.2	67.5	12.0	12.8	18.4	36.4
Primary	69.4	71.4	12.9	12.8	27.5	24.8
Secondary	63.9	65.2	12.3^d^	11.8^d^	29.7	31.3
Tertiary	64.9	66.3			36.3	35.8
Age group						
<25	73.6	74.6	14.1	16.7	20.8	25.5
25-34	71.4	72.4	14.4	14.2	28.0	28.4
35-49	48.1^c^	50.3^c^	10.5	10.5	35.0^a^	35.9
Parity						
0-1	64.9	51.8^b^	8.8	10.4	21.3	23.8
2-3	69.8	70.3	13.5	13.8	26.7	29.4
4+	65.1	66.7	13.7	12.6	38.5^c^	36.4
Polygyny						
No	67.3	67.6	13.2	13.1	28.4	29.8
Yes	63.0	69.0	5.4	6.7	31.9	32.3
Wealth						
Lowest	73.9	73.9	14.2	15.3	21.3	26.1
Lower	69.2	70.4	16.6	14.3	29.4	29.0
Middle	71.6	72.5	12.2	13.5	33.1	30.9
Higher	63.6	63.2	8.8	11.6	32.0	35.4
Highest	56.4^b^	57.1^b^	11.5	9.1	31.6	31.1

^a^ *p* < 0.05; ^b^ *p* < 0.01; ^c^ *p* < 0.001; ^d^Secondary/Tertiary

Tables 2 and 3 reveal information on change in fertility desires and modern contraceptive use between baseline and follow up among women with complete data. As shown in Table 2, the magnitude of change in fertility desires was comparable across the three study sites. About one in five women who wanted more children at baseline reported they wanted no more children at follow up. Slightly lower proportions of women at follow up shifted fertility desires in the opposite direction, with a range of 15.7 to 19.1 % wanting no more at baseline and more at follow up.

**Table 2:** Change in fertility desires between baseline and follow up by site

Fertility desire		Sebeta, Ethiopia		Asawase, Ghana		Ipetumodu, Nigeria	
		FOLLOW UP					
		Want more	No more	Want more	No more	Want more	No more
BASELINE	Want more	78.1	21.9	77.0	23.0	77.6	22.5
	No more	18.6	81.4	15.7	84.3	19.1	80.9

**Table 3:** Change in modern contraceptive use between baseline and follow up by study site

Modern contraceptive use status		Sebeta, Ethiopia		Asawase, Ghana		Ipetumodu, Nigeria	
		FOLLOW UP					
		Non-users	Users	Non-users	Users	Non-users	Users
BASELINE	Non-users	66.5	33.5	91.5	8.5	69.6	30.4
	Users	25.0	75.0	73.4	26.6	47.6	52.4

Table 3 shows that 75.0 %, 26.6 % and 52.4 % of users in Sebeta, Asawase and Ipetumodu, respectively, reported using a modern method both at baseline and follow up. In the same order, 66.5 %, 91.5 % and 69.6 % of women in these sites reported consistent non-use of a modern method at both time points. Change in modern contraceptive use status was greatest in Asawase where almost three-quarters of women who reported use at baseline were no longer using a modern method at follow up. Change in the other direction from non-use at baseline to use of a modern method at follow up was greatest in Sebeta (33.5 %), followed by Ipetumodu (30.4 %).

Table 4 shows the results of multivariate GLM analysis. The table presents findings from two models for each of the three peri-urban sites. The first model is unadjusted for potential confounders and includes the main independent variable, fertility desires, the time variable, and an interaction between the two. The second model is adjusted for age of the woman, parity, highest educational level attained, wealth quintile and polygyny. At baseline and for all sites, women who wanted no more children had higher odds of contraceptive use compared with those who wanted more children. However, although the odds ratios were in the hypothesized direction, they did not reach statistical significance in Asawase. The adjusted odds ratios (AOR) of modern contraceptive use comparing follow up to baseline among women who wanted more children, (X = 0) were 0.66 (95 % CI 0.51, 0.85) and 0.49 (95 % CI 0.28, 0.84) in Sebeta, Ethiopia, and Asawase, Ghana, respectively, but this did not attain statistical significance in Ipetumodu, Nigeria (AOR 1.21 (95 % CI 0.86, 1.72)). The relative odds of modern contraceptive use comparing follow up to baseline among women who wanted no more children (X = 1) were 0.87 (95 % CI 0.64, 1.17), 1.26 (95 % CI 0.80, 1.97) and 1.55 (95 % CI 1.07, 2.26) in Sebeta, Asawase and Ipetumodu respectively, attaining statistical significance only in Ipetumodu.

**Table 4:** Adjusted ORs and 95 % CIs showing influence of change in fertility desires on change in modern contraceptive use

Covariate	ETHIOPIA (UNADJ)	ETHIOPIA (ADJ)^d^	GHANA (UNADJ)	GHANA (ADJ)^d^	NIGERIA (UNADJ)	NIGERIA (ADJ)^d^
Fertility desire at baseline (Ref = Want more)	1.40 (1.06, 1.85)^a^	2.03 (1.44, 2.85)^c^	1.18 (0.76, 1.84)	1.30 (0.77, 2.19)	1.71 (1.23, 2.38)^c^	1.60 (1.06, 2.41)^a^
Time (Ref = Baseline)	0.68 (0.54, 0.86)^c^	0.66 (0.51, 0.85)^c^	0.49 (0.28, 0.84)^a^	0.49 (0.28, 0.84)^a^	1.28 (0.92, 1.79)	1.21 (0.86, 1.72)
Baseline fertility desire X Time	1.32 (0.92, 1.90)	1.32 (0.89, 1.97)	2.71 (1.31, 5.62)^a^	2.59 (1.24, 5.41)^a^	1.13 (0.70, 1.85)	1.28 (0.77, 2.13)
Time + Baseline fertility desire X Time	0.90 (0.69, 1.18)	0.87 (0.64, 1.17)	1.32 (0.85, 2.05)	1.26 (0.80, 1.97)	1.45 (1.02, 2.07)^a^	1.55 (1.07, 2.26)^a^
Constant	1.77 (1.50, 2.09)^c^	0.82 (0.48, 1.39)	0.14 (0.10, 0.18)^c^	0.07 (0.03, 0.17)^c^	0.35 (0.28, 0.43)^c^	0.07 (0.03, 0.19)^c^

^a^ *p* < 0.05; ^b^ *p* < 0.01; ^c^ *p* < 0.001

^d^Models were adjusted for age, parity, educational level, wealth and polygyny

Ethiopia: Sebeta; Ghana: Asawase; Nigeria: Ipetomodu

(For these generalized linear models with an underlying binomial distribution, an exchangeable correlation structure was assumed and robust variances estimated, using maximum likelihood estimation.)

X: multiplication

## Discussion

In the peri-urban settings we studied, we found that change in women’s fertility desires was associated with change in their contraceptive behavior. As hypothesized, at both time points, women who wanted no more children reported more contraceptive use compared with those who wanted more children in all three peri-urban sites. Our findings with longitudinal data strengthen those from cross-sectional studies that find women’s fertility desires or intentions are associated with their report of contraceptive use [4, 5]. It is possible that there is some threshold of community-level contraceptive use above which this finding holds true, given the absence of a significant association in Asawase where contraceptive use was lowest. However, this hypothesis would need to be tested in further studies involving several other peri-urban settings.

Our hypothesis that women who wanted more children at baseline would report less contraceptive use at follow up held true in Sebeta and Asawase, but not in Ipetumodu. The overall increase in modern contraceptive use in Ipetumodu may account, at least in part, for the unexpected finding whereby women who wanted more children reported an increase in contraceptive use over time. This suggests an inconsistency between fertility desires and fertility behavior in the Ipetumodu cohort. Another potential reason for this finding may be the commitment of the Nigerian Federal Government to family planning which led to the introduction of contraceptive commodities as a budget line item in 2012 and the availability of free commodities in government-owned family planning clinics. It is plausible that the increased access to modern contraception can account for this overall increase in contraceptive use among all women in Ipetumodu, irrespective of their reported fertility desires.

Although we hypothesized that women who wanted no more children at baseline would report more contraceptive use at follow up, compared to baseline, this finding only attained statistical significance in Ipetumodu, with the relationship holding true even after adjusting for confounders. Although the reason for this is not clear, women in this peri-urban site may have been more motivated than their counterparts in Sebeta and Asawase. It is possible that the reversal of the expected direction of the effect observed in Sebeta was an underestimate of the effect, due to some unmeasured confounding. The widespread use of community health extension workers, political will, donor funding and the nongovernmental and public–private partnerships are believed to account for Ethiopia’s family planning success story, [14] reflected in the high prevalence of modern contraceptive use in this peri-urban community.

Relatively high attrition in the sample followed up was an important limitation of this study, like most longitudinal studies. A key strength of this study was the peri-urban, SSA setting for which little published data exists. A further strength was the availability of data from two time points for the same women. While the findings of this study cannot be extended beyond maritally stable and co-residential women in the respective study sites, it is worth noting that per the 2013 Nigeria Demographic and Health Survey [13] modern contraceptive use among currently married women was higher in Osun state, where Ipetumodu is located, than elsewhere in the country. Thus, program and development factors may be at play in Nigeria, as well as in Ethiopia and Ghana, enabling urban women to translate their fertility desires and intentions into contraceptive behavior. The implications of our findings are that reducing the psychosocial and financial costs of contraception may make a difference for positive change in contraceptive uptake. This has been revealed by the national success of the family planning program in Ethiopia [14] and the emerging increase in contraceptive use in the Nigerian peri-urban community studied. Findings differed by site, highlighting that context matters. Mixed methods studies can also provide more insight into observed differences across contexts and assist in framing and planning future interventions.

## Conclusions

In the three peri-urban sites studied, women's self-reported fertility desires influenced their contraceptive behaviour over time, with context-specific differences in the pattern of change observed. In Ipetumodu, Nigeria, women who wanted no more children reported increased use of contraception, while in Sebeta, Ethiopia, and Asawase, Ghana, women who wanted more children reported less contraceptive use over time. Existing programs need to understand the dynamics of changing contraceptive demand so as to ensure the services they offer remian flexible enough to enable women in peri-urban settings to act on their fertility desires.
